# Prediction of *C. elegans* Longevity Genes by Human and Worm Longevity Networks

**DOI:** 10.1371/journal.pone.0048282

**Published:** 2012-10-29

**Authors:** Robi Tacutu, David E. Shore, Arie Budovsky, João Pedro de Magalhães, Gary Ruvkun, Vadim E. Fraifeld, Sean P. Curran

**Affiliations:** 1 The Shraga Segal Department of Microbiology, Immunology and Genetics, Center for Multidisciplinary Research on Aging, Ben-Gurion University of the Negev, Beer-Sheva, Israel; 2 Department of Molecular Biology, Massachusetts General Hospital, Boston, Massachusetts, United States of America; 3 Department of Genetics, Harvard Medical School, Boston, Massachusetts, United States of America; 4 Integrative Genomics of Ageing Group, Institute of Integrative Biology, University of Liverpool, Liverpool, United Kingdom; 5 Division of Biogerontology, Davis School of Gerontology, University of Southern California, Los Angeles, California, United States of America; 6 Department of Molecular and Computational Biology, Dornsife College of Letters, Arts, and Sciences, University of Southern California, Los Angeles, California, United States of America; 7 Department of Biochemistry and Molecular Biology, Keck School of Medicine, University of Southern California, Los Angeles, California, United States of America; Albert Einstein College of Medicne, United States of America

## Abstract

Intricate and interconnected pathways modulate longevity, but screens to identify the components of these pathways have not been saturating. Because biological processes are often executed by protein complexes and fine-tuned by regulatory factors, the first-order protein-protein interactors of known longevity genes are likely to participate in the regulation of longevity. Data-rich maps of protein interactions have been established for many cardinal organisms such as yeast, worms, and humans. We propose that these interaction maps could be mined for the identification of new putative regulators of longevity. For this purpose, we have constructed longevity networks in both humans and worms. We reasoned that the essential first-order interactors of known longevity-associated genes in these networks are more likely to have longevity phenotypes than randomly chosen genes. We have used *C. elegans* to determine whether post-developmental inactivation of these essential genes modulates lifespan. Our results suggest that the worm and human longevity networks are functionally relevant and possess a high predictive power for identifying new longevity regulators.

## Introduction

Numerous pathways contribute to longevity, but the identification of their components has not been saturating [1]. Because of their short lifespan and genetic tractability, *C. elegans* have proven indispensable in the study of longevity. The first screen to identify *C. elegans* genes that regulate longevity was an EMS mutagenesis that isolated eight mutants, each of which modulated the dauer developmental pathway or caloric intake [2]. The relationship between these functions and lifespan is now well established [3], [4]. Two subsequent studies utilized genome-wide RNA interference (RNAi) to identify genes that increase longevity when inactivated [5], [6]. These screens identified 89 and 29 genes respectively, with an overlap of only 3 genes, strongly suggesting that neither was saturating. This likely reflects the high false negative rate associated with high-throughput RNAi screening, as well as technical limitations of the screen designs [1]. For instance, because the screens inactivated genes of interest during development, genes required for development but capable of modulating adult lifespan would be missed. Curran and Ruvkun explored this overlooked gene set by inactivating essential genes postdevelopmentally, revealing 64 genes required for development that extend lifespan when inactivated during adulthood [7]. Nevertheless, many important longevity genes likely remain unidentified.

**Figure 1:**
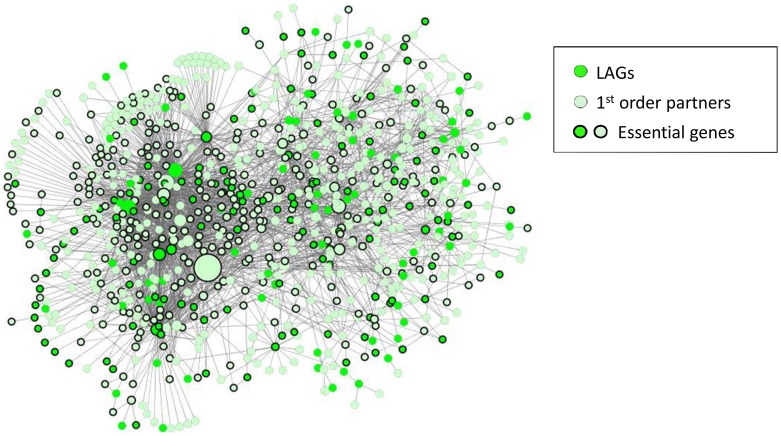
Worm Longevity Network. The worm longevity network (WLN) includes 205 previously identified LAGs (dark green) and their 666 first-order protein interaction partners (light green). The graphical output of the network was generated using Cytoscape 2.8.0 [51]. Size of nodes is proportional with the number of PPIs in the BioGRID interactome. Genes in the network are more connected and more interconnected than would be expected by chance, suggesting shared functionality. Because many genes that influence longevity function in complexes, signaling networks or in conjunction with cofactors, the 666 first-order interacting LAG partners may be enriched with previously unidentified longevity genes.

Known longevity genes are enriched for roles in stress tolerance and development. Many mutations that modulate longevity were identified by virtue of their contribution to stress response pathways or by homology to genes of this kind. A study of over 50 long-lived *C. elegans* mutants reveals that each is resistant to one or more stressors, such as oxidative damage, heat stress or irradiation [8], [9]. Many genes required for the successful extension of lifespan in one or more long-lived mutants also contribute to the longevity of wild-type animals, but are distinguished by a proportionally greater change in the mutant background. Examples of such genes include *daf-16*, *hsf-1*, *smk-1*, *jnk-1*, *cst-1*, *skn-1*, and *pha-4* [10], [11], [12], [13], [14], [15]. Overexpression of most of these genes extends lifespan and, where tested, increases stress tolerance. Network analyses have also revealed a link between aging-related genes and development; known longevity-associated genes (LAGs) are enriched for essential genes or those required for development, and essential genes are likewise enriched for LAGs [7], [16], [17], [18], [19]. This finding appears to illustrate the antagonistic pleiotropy theory of aging, which suggests that the post-reproductive decrescendo of the force of natural selection permits the evolution of genes that are essential early in life but detrimental late in life [20].

**Table 1:** First-order interactors of LAGs in the WLN regulate lifespan in *C. elegans.*

Gene	Common name	Lifespan (%Δ mean)^a^	Lifespan (%Δ max)^a^	Function
T23D8.3	T23D8.3	25.7	34.7	Translation
F37C12.9	*rps-14*	16.6	21.7	Translation
T09A5.10	*lin-5*	16.4	8.7	Cell division
C03C10.3^b^	rnr-2	14.2	21.7	DNA biosynthesis
F54E7.2	*rps-12*	11.6	21.7	Translation
F09F7.3	F09F7.3	8.1	21.7	RNA polymerase III
C38D4.3	*mel-28*	6.5	8.7	Cell division
T20B12.8	*hmg-4*	6.5	0.0	Transcription elongation
F35G12.10	*asb-1*	6.4	0.0	ATP synthase
F26F4.11^b^	rpb-8	6.4	0.0	RNA polymerase II
C47D12.2	C47D12.2	5.5	8.7	Unknown
K10B3.7	*gpd-3*	5.3	0.0	Glycolysis
R144.2	R144.2	−18.9	−25.0	mRNA cleavage and polyadenylation
K07D4.3	*rpn-11*	−19.3	−25.0	Proteasome
B0285.1	B0285.1	−22.2	−25.0	Kinase
R13G10.1	*dpy-27*	−23.5	−25.0	Condensin
W07B3.2	*gei-4*	−27.0	−38.0	Filament regulation

a %Δ mean and %Δ maximum lifespan (last quartile) were calculated in relation to control.

b Shared genes in WLN and HLN.

Samuelson et al. (2007) screened for gene inactivations that suppress lifespan extension in *daf-2* mutant *C. elegans* and identified 159 genes contributing to *daf-2* lifespan and to stress tolerance [21]. The majority of the suppressors decrease the longevity of a control strain, but decrease *daf-2* longevity by a greater margin. Based upon the efficacy of other genome-wide screens and technical limitations, it is unlikely that this screen saturated the breadth of genes that contribute to lifespan extension.

Network biology is one approach to gaining insight regarding the interactions of known LAGs and identifying new longevity regulators [22], [23]. Network approaches provide a conceptual framework for the study of the complex interactions amongst the components of biological systems [24]. Networks may be constructed from many kinds of data, including, but not limited to, protein-protein interactions, transcriptional co-regulation, putative microRNA targets, or participation in annotated biological pathways [16], [18], [25]. Databases of such interactions exist for many species including yeast, worm, fly, mouse, and human [26]. Often, genes that serve essential cell functions are more connected than others and genes that contribute to a particular phenotype are more interconnected than would be expected by chance [19], [22], [27].

**Table 2:** First-order interactors of LAGs in the HLN regulate lifespan in *C. elegans*.

Gene	Common name	Lifespan (%Δ mean)^a^	Lifespan (%Δ max)^a^	Function
C03C10.3^b^	rnr-2	14.2	21.7	DNA biosynthesis
R02D3.3	R02D3.3	13.0	21.7	RNA polymerase II
F10B5.6	emb-27	10.5	8.7	Cell division
F29G9.3	aps-1	7.9	0.0	Adaptin
T09B4.10	chn-1	7.1	8.7	Ubiquitin ligase
F26F4.11^b^	rpb-8	6.4	0.0	RNA polymerase II
F28D9.1	rsr-1	6.1	0.0	Splicing
F18A1.5	rpa-1	5.1	21.7	DNA replication
C01H6.5	nhr-23	4.9	8.7	Molting
Y40B1A.4	sptf-3	−22.9	−37.0	Transcription factor
ZK1058.2	pat-3	−28.1	−50.0	Integrin
C52E4.4	rpt-1	−28.4	−50.0	Proteasome
F26H9.6	rab-5	−38.0	−50.0	Endocytosis
D1014.3	snap-1	−39.9	−50.0	Vesicle fusion
K02D10.5	K02D10.5	−48.6	−72.0	SNARE complex

a %Δ mean and %Δ maximum lifespan (last quartile) were calculated in relation to control.

b Shared genes in WLN and HLN.

Previous network analyses have demonstrated that LAGs, on average, have more protein-protein interactions (PPIs) with other proteins and amongst each other than non-LAGs in the interactome [28]. This is consistent with the fact that many LAGs play significant roles in development, participate in complex stress response cascades or are otherwise essential. Given the wealth of LAGs, an effort to understand the regulation of longevity from a biological network perspective may provide new insights into longevity pathways.

Networks may be enriched by the integration of information from diverse species using homology as a means to overlay species-specific findings [26]. This technique could be applicable to aging because LAGs are highly conserved across species [7], [22], [28]. Such an approach may be especially fruitful in the study of human aging because aging has been extensively studied in model organisms. The profusion of data from non-mammalian systems renders broader analyses increasingly powerful and informative. An interaction map enriched with data across all species and accounting for cross-species homology could generate a robust functional network and be used to identify new genes in lifespan extension pathways.

In this study, we performed a network analysis of LAGs and their interacting partners in worms and humans. We found that LAGs and their first-order partners form tightly interconnected networks. The partners of known LAGs in the worm and human longevity networks may participate in the intricate pathways and complexes that regulate lifespan, and are therefore candidate longevity genes. Essential genes are particularly interesting in this regard because known LAGs are enriched for developmental functions, consistent with the concept of antagonistic pleiotropy [7], [16], [22]. To functionally verify this prediction, we post-developmentally inactivated 374 of these genes or their orthologs in *C. elegans*. In our primary analysis, 156 of these inactivations resulted in extended (101) or decreased (55) lifespan. We confirmed a subset (30 genes) of these phenotypes in rigorous longitudinal analyses. Our results are consistent with the idea that genes involved in development and translation have a role in longevity regulation. Collectively, this study presents a proof of concept that by combining a network-based approach with the selection of genes fitting antagonistic pleiotropy, new worm lifespan regulators could be identified with an unprecedentedly high predictive power.

**Table 3:** A network-based approach verifies new worm longevity genes at a greater frequency than genome-wide RNAi screens.

Gene set	Phenotype	Genes screened	Preliminary candidates	Retested	Verified	Frequency^a^
Genome-wide^[6]^	Long-lived	16475	600	600	89	0.54
Genome-wide^[5]^	Long-lived	13300	94	94	29	0.22
Essential genes^[7]^	Long-lived	2700	470	470	64	2.37
Genome-wide^[21]^	Short-lived	15718	500	500	159	1.01
LAG interacting partners(this study)	Long-lived	374	101	45	19	5.08
	Short-lived	374	55	12	11	2.94

a The frequency with which longevity genes were verified in each screen is presented as a percentage of the total number of genes screened.

## Materials and Methods

### Data Sources

*C. elegans* LAGs were compiled from scientific literature and manually curated. The list of LAGs includes genes reported to promote longevity or cause premature aging following genetic intervention (partial or full loss-of-function mutations, gene overexpression) or RNA interference-induced gene silencing [17], [22], [29]. The collection contains 555 entries and is accessible in the Human Ageing Genomic Resources – GenAge database (build 14), http://genomics.senescence.info/genes/index.html [29]. This list of genes was used as a “core set” for the construction of the worm longevity network (WLN).

In addition to established human LAGs – the number of which is still very limited – the “core set” for the construction of human longevity network (HLN) also included non-redundant orthologs of LAGs from model organisms (*S. cerevisae*, *C. elegans, D. melanogaster*, and *M. musculus*). In total, this list consists of 662 human genes. All LAG lists for model organisms were compiled using the same method described for *C. elegans* and are available in the GenAge database [29].

**Table 4:** Distribution of long-lived and short-lived phenotypes and the frequency of verified longevity regulators (LAGs) in this study.

Gene set	Genes screened	Phenotype	Preliminary candidates	Retested	Verified	Frequency^a^
WLN^b^	147	Long-lived	47	24	10	6.8
		Short-lived	21	5	5	3.4
HLN^b^	184	Long-lived	45	17	7	3.8
		Short-lived	27	6	6	3.3
Shared	43	Long-lived	9	4	2	4.7
		Short-lived	7	1	0	0.0
Total	374	Long-lived	101	45	19	5.1
		Short-lived	55	12	11	2.9

a The frequency with which longevity genes were verified in each gene set is presented as a percentage of the total number of genes screened.

b WLN and HLN without shared genes.

PPI data used for the constructions of WLN and HLN were extracted from the BioGRID database, release 2.0.53 [30]. Orthology information was obtained from the InParanoid database – Eukaryotic Ortholog Groups, release 6.1 [47] and worm lethal phenotypes were retrieved from the WormBase database [48], [49].

### Network Construction

The approach for constructing the longevity networks was described in detail elsewhere [22], [28]. The longevity networks for worms and humans were created using YABNA (Yet Another Biological Networks Analyzer), a flexible software program developed in Vadim Fraifeld’s lab. Current versions of human and model organism longevity networks are available in the NetAge database [28]. For both worm and human gene sets, the network construction algorithm included: 1) keeping all genes (LAGs) with reported PPIs from the “core set”; 2) adding all first-order PPI partners of core genes; and 3) taking the largest interconnected sub-graph as a longevity network.

### Prediction of New Worm Longevity Regulators Based on WLN/HLN

Prediction of new LAGs in *C. elegans* was based on all the following criteria: 1) belonging to the WLN or being the *C. elegans* ortholog of a gene from the HLN; 2) not reported previously as a LAG in *C. elegans* or other model organisms; 3) being essential for the development and growth of *C. elegans* (essential genes). Thus, there were two sources for selection of candidate genes―the first-order partners of LAGs from WLN and HLN. In total, 500 essential worm genes were included as candidates for longevity analysis. Two hundred twenty-eight of these genes were derived from the WLN and the remainder from the HLN, with an overlap of 54. RNAi clones were available to target 374 of these 500 genes.

**Figure 2:**
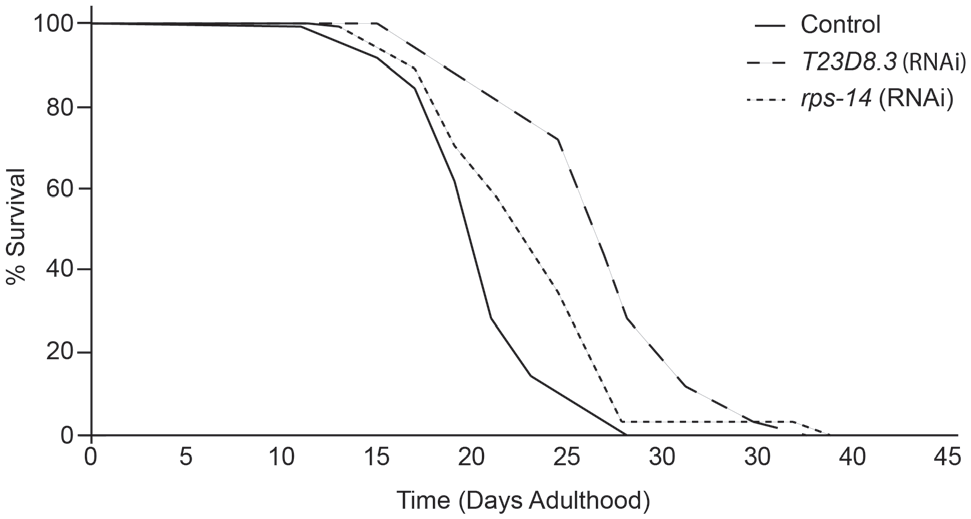
Disruption of translation extends longevity in *C. elegans*. The gene inactivations found to extend lifespan by the greatest percentage function in translation. T23D8.3 is the *C. elegans* ortholog of human LTV1, which is required for the nuclear export and processing f the 40S ribosomal subunit. The ribosomal protein subunit *rps-14* directly participates in the 40S ribosome and is required for translation. Mean lifespan extension following postdevelopmental inactivation of these genes in an enhanced RNAi strain, *eri-1*, is 26% for the LTV1 ortholog (dashed line) and 16.6% for *rps-14* (dotted line) in comparison to an empty vector RNAi control (solid line). The lifespan extension phenotypes of these genes are consistent with the phenotypes of other translation genes in our screen, including *rps-12* and 3 RNA polymerases (Tables 1 and 2).

### Detection of Functional Enrichment

To detect enriched functions and processes, the Database for Annotation, Visualization, and Integrated Discovery (DAVID) was employed using default settings [50]. For detecting functional enrichment in the genes tested, the worm genome was used as background. For detecting enrichment in genes resulting in a short- or long-lived phenotype, the genes tested were used as background.

**Figure 3:**
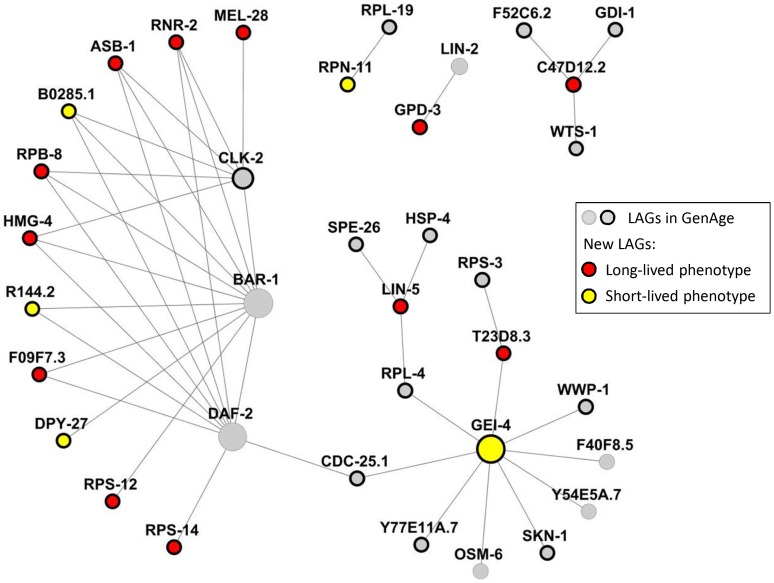
Interaction of new LAGs with known worm longevity genes. The LAGs identified in our screen interact extensively with known longevity genes. Nearly two-thirds of these genes interact with *daf-2* and *bar-1*, both involved in the *daf-16*-mediated lifespan extension, and *clk-2*, a gene whose mutations may extend lifespan by slowing down development. Size of nodes is proportional with the number of PPIs in the BioGRID interactome. Bordered circles depict genes essential for growth and development.

### Strains and Culture Conditions

*C. elegans* were cultured on *Escherichia coli* OP50 or HT115 by standard techniques. *C. elegans* N2 Bristol (WT) and GR1373 *eri-1*(mg366) IV were utilized in the described experiments.

### Post-developmental RNAi

Post-developmental RNAi lifespan analysis was performed as described by Curran and Ruvkun, 2007 [7]. RNAi clones were grown overnight in LB with carbenicillin and seeded to 6-well plates containing 5 mM isopropylthiogalactoside (IPTG). 400 µl bacteria were seeded to each well. Expression of double-stranded RNA was induced overnight. *eri-1(mg366)* worms raised to the L4 larval stage on HT115 *E. coli* were washed twice in M9 buffer with tetracycline, carbenicillin and streptomycin, resuspended in M9 carbenicillin buffer, and seeded onto the RNAi induced bacteria. Wells were treated with 80 µg/ml 5-fluorodeoxyuridine (FUDR) to inhibit progeny production. On the third day of adulthood, wells were supplemented with 50 µl of 10x concentrated RNAi bacteria grown overnight and induced for two hours at room temperature in LB with carbenicillin and 5 mM IPTG.

### Survival Analysis

Post-developmental gene inactivation was carried out as described above. Primary survival analysis was accomplished by a thrashing assay in which replicate wells were flooded with M9 buffer on the 15^th^, 17^th^, 19^th^ and 21^st^ days of adulthood at 20°C [21]. One of four replicates was scored and subsequently discarded for each time point. Preliminary survival phenotypes were assigned to gene inactivations that produced a consistent 10% increase or decrease in survival on at least three of the four time points. Strong candidates were selected for longitudinal lifespan analysis. In each biological replicate and at each time point in the thrashing assay, at least 30 worms were scored for each condition.

### Adult Lifespan Analysis

From the four biological replicates in the thrashing assay we selected RNAi clones that consistently yielded the strongest phenotypes (both long and short lived) at each of the time points screened. Post-developmental gene inactivation was carried out as described above. Longitudinal lifespan analyses were conducted as described with incubation at 20°C or 25°C. Survival was assayed by gently tapping worms with a thin length of platinum every other day from young adulthood onward. Statistical analyses were performed using the software SPSS (SPSS Inc.). Survival of each RNAi-treated population was compared with that of a population treated with control RNAi, and significance was determined by Kaplan-Meier analysis using the log-rank test. A significance threshold of p<0.05 was applied. On average, 90 animals were scored longitudinally across three biological replicates for each clone.

## Results and Discussion

The *C. elegans* LAG set contains 555 previously identified positive and negative regulators of longevity [29]. Of these, 218 are present in the *C. elegans* interactome in the BioGRID database [30]. These LAGs display a higher degree of connectivity than would be expected by chance and more importantly, a very high degree of interconnectivity. Almost a quarter of worm LAGs found in the interactome are interconnected, whereas simulations with randomized gene sets of the same size predict only 5% interconnectivity (p<0.001). Because PPI databases are inherently incomplete, the distribution of interactions may change as entries accumulate. This does not detract from the significance of individual PPIs currently found within the database.

To exploit LAG connectivity as a means to identify new candidate longevity genes, we explored the first-order partners of *C. elegans* LAGs. Taken together with their first-order partners, 94% of the LAGs in the interactome form a continuous Worm Longevity Network (WLN) (Figure 1). The network encompasses 871 genes, of which 205 are known LAGs at the core of the network and 666 are their first-order partners not previously associated with longevity. Because many longevity genes function within complex signaling pathways or functional groups, we propose that the 666 first-order partners of LAG network genes are likely to include factors that regulate longevity.

To verify the functional significance of LAG PPIs, we inactivated first-order partners of LAGs and assayed their effect on lifespan in *C. elegans*. We focused our search on a subset of 228 of the 666 first-order partners, selected because they are essential in development, a process closely associated with longevity regulation [7], [17], [18], [19], [28]. RNAi clones were available for 190 of these 228 genes (Tables S1 and S3). Evidence that previous screens have not been saturating supports the possibility that a rigorous analysis of our systematically selected candidate gene set will reveal previously unidentified LAGs.

Because LAGs are highly evolutionary conserved, we also utilized a Human Longevity Network (HLN) to identify candidate regulators of lifespan [17], [22], [28]. The HLN was previously constructed by compiling a set of genes directly associated with human aging, such as those responsible for progeria, and the orthologs of LAGs experimentally defined in other species [18], [22]. Together with their first-order partners, these genes form a continuous PPI network [22], [28]. Like the first-order partners of the WLN, the first-order partners of HLN LAGs could also be considered putative new longevity regulators. As human studies to test network predictions experimentally are not possible, we elected to test the *C. elegans* orthologs of the first-order partners of LAGs in the HLN. We further narrowed our candidate list from the HLN to 272 orthologs previously found to be required for development in *C. elegans*. RNAi clones targeting 227 genes were available (Tables S2 and S3). Forty-three of the *C. elegans* orthologs of HLN genes with available RNAi clones were already present amongst the 190 WLN candidates, suggesting that the first-order interactions of LAGs, like the genes themselves, may be highly conserved.

We chose to inactivate the 374 putative *C. elegans* LAGs identified by the above network analysis post-developmentally (Tables S1–S3). This approach was necessitated by our focus upon genes required for development, an ontology enriched with known LAGs. Post-developmental gene inactivation circumvents developmental pleiotropies, thus simplifying the interpretation of longevity phenotypes. Genes essential for development are enriched approximately 5-fold for LAGs and 15-fold for gene inactivations that extend lifespan by greater than 20% [5], [6], [7]. In addition, evidence suggests that many lifespan regulatory treatments that are effective when applied during development are also effective if initiated during adulthood [7], [31], [32], [33], [34], [35]. Therefore, post-developmental gene inactivation maximizes our capacity to detect genes that regulate longevity.

In our primary screen consisting of the post-developmental inactivation of 374 candidate LAGs, lifespan was approximated by survival at four time points distributed across the period of mortality. In this primary analysis, 101 gene inactivations conferred a consistent increase in survival and 55 a decrease, compared to controls (Tables S1–S3). To confirm these results in a more rigorous analysis, we selected 57 novel putative LAGs (45 long-lived and 12 short-lived phenotypes) for longitudinal analysis on the basis of phenotypic strength. Longitudinal analysis confirmed 19 of the gene inactivations that increase lifespan and 11 that decrease lifespan, thus identifying 30 new *C. elegans* LAGs (Tables 1 and 2). The rate of identification for lifespan-promoting gene inactivations (19/374; 5.1%) is much greater than that achieved in genome-wide screens (average 0.38%), as well as that of an unbiased screen that canvased genes known to play critical roles in development (2.4%, Tables 3 and 4) [5], [6], [7]. This comparison is crude because conditions and selection criteria utilized in each screen were distinct. However, if the rate at which our preliminary results were confirmed longitudinally could be extrapolated to the untested preliminary hits, the set of 374 candidate LAGs would include 42 (11.2%) that increase lifespan when inactivated and 50 (13.4%) that decrease it. This is likely to be an overestimate, however, because the genes selected for secondary analysis represent the strongest preliminary scores.

Most of the longitudinally confirmed gene inactivations that increased mean lifespan did so by a small but statistically significant percentage when performed post-developmentally (average 9.7%). Previous screens for increased lifespan have identified numerous genes that extend lifespan by upwards of 20%. In this screen, we report only one gene at that level, *T23D8.3*, which extends mean lifespan by 26% (Table 1, Figure 2). *T23D8.3* is required for embryonic and larval development. This is also true of its ortholog, LTV1, which is essential for cell growth in yeast and cultured human cells [36]. LTV1 participates in the nuclear export and processing of the 40S ribosomal subunit, suggesting that T23D8.3 inactivation inhibits translation in *C. elegans*. The second greatest extension of mean lifespan resulted from inhibition of a protein subunit of the 40S ribosome (*rps-14*), another component of translation, with 16.6% extension of mean lifespan (Table 1, Figure 2).

The identification of gene inactivations with modest lifespan extension phenotypes (12 of 19 gene inactivations <10%, Tables 1 and 2) is consistent with our hypothesis in several ways. First, we predicted that we would identify gene inactivations missed in previous screens (false negatives). The subtlety of some of the observed phenotypes may explain, in part, how they were overlooked. Second, previous LAG network studies have highlighted the existence of nodes representing key lifespan regulatory factors [16], [37]. Our results suggest that genes interacting directly or indirectly with these nodes, such as those tested here, contribute to their activity, perhaps additively, but are not strictly required.

We performed a functional enrichment analysis using DAVID to determine whether translation or other processes were enriched within our results. In total, 17 of the gene inactivations that conferred increased longevity in our preliminary analysis are involved in translation, including initiation factors, tRNA synthetases, and ribosomal subunits. This ontology was not present for genes found to decrease lifespan when inactivated. Therefore genes required for translation are specifically enriched amongst those that extend lifespan when inactivated in comparison to both the set of genes tested (p = 4.1E-4) and the *C. elegans* genome as a whole (p = 3.3E–8). Previous studies have demonstrated that translation inhibition is a potent mechanism of longevity extension [7], [38], [39], [40], [41]. Current models of longevity extension thereby substantiate the results of our combined *in silico* and *in vivo* analyses.

We longitudinally confirmed 11 positive regulators of lifespan; these genes decrease *C. elegans* longevity when inactivated post-developmentally. The frequency at which these genes were identified (11/374, 2.9%) is comparable to that achieved in a previous genome wide screen for positive regulators of lifespan in *daf-2* mutants (3.2%) [21]. Our results, however, represent the first systematic analysis of short-lifespan phenotypes in a wild-type background. The actual number of positive regulators in our candidate LAG set may have been significantly greater than 11 because 92% of candidates retested were confirmed and 44 preliminary candidates were not retested. Mutation or inactivation of many genes with key longevity-regulatory functions reduces wild-type longevity while abrogating lifespan extension in one or more long-lived mutants, resulting in the equalization of wild-type and mutant lifespans [10], [11], [12], [13], [14], [15]. The genes identified in this screen are particularly intriguing because they interact with known components of the LAG network. It would be interesting to determine whether the positive regulators we have identified contribute specifically to particular mechanisms of lifespan extension, such as insulin/IGF-1 signaling or the disruption of translation. Determining the molecular roles these genes might play in lifespan extension will be a topic of continued research.

Analyzing the interactions of the LAGs identified in this screen within the existing WLN reveals that almost two thirds of the new LAGs are connected to at least one of the prominent lifespan regulatory genes *daf-2*, *bar-1*, or *clk-2*. Moreover, 5 genes (*rnr-2*, *asb-1*, *rpb-8*, *hmg-4*, and B0285.1) interact with all 3. Multiple connections to established LAGs were also observed for *gei-4*. The WLN LAGs identified in this screen and their first-order LAG partners form a continuous network – a WLN module, which is presented in Figure 3.

We separated results from the *WLN* (Table S1), the *C. elegans* orthologs from the HLN (Table S2) and genes common to both sets to determine the functional efficacy of each selection (Table S3). For this analysis, we considered only the primary results because the number of genes selected for inclusion in the longitudinal analysis was independent of these groupings. Results from each class are similar. Primary analysis detected lifespan phenotypes for 46% (32% long-lived, 14% short lived) of WLN-specific candidates (Table S1), 39% (24% long-lived, 15% short-lived) of HLN-specific candidates (Table S2) and 37% (21% long-lived and 16% short-lived) of the candidates common to both groups (Table S3). Although the modest increase in efficacy with which the WLN predicted increased lifespan may indicate superior accuracy, failure to replicate that observation amongst shared LAGs suggests this is not the case. Importantly however, the orthologs of human interactors were similarly predictive with the interactors endogenous to *C. elegans*, underscoring the remarkable conservation of genes that regulate longevity as well as their protein-protein interactions.

We confirm 30 new LAGs identified through network analysis of *C. elegans* and human candidates. Our results are consistent with several empirical observations regarding genes found to regulate longevity in previous screens. First, in agreement with previous studies, we identify a strong association of developmental and lifespan-regulatory functions [7], [17], [18], [19], [28], [42], [43], [44], [45]. By utilizing PPI networks and applying developmental ontology filters, we identified new lifespan regulators with a 13-fold greater frequency than has been reported in previous genome-wide screens (5.08% of long-lived phenotypes vs. an average of 0.38% in two genome-wide screens [5], [6]; Tables 3 and 4). This is particularly supportive of our approach because we excluded all previously identified LAGs, suggesting we achieved greater efficacy despite systematically omitting the most robust, and therefore most easily identified, LAG inactivations. The verification of new LAGs resulting from our analysis probably underestimates the total present in our candidate pool, as 56 gene inactivations that extend lifespan and 43 that decrease lifespan from our preliminary analysis were not retested longitudinally. Second, the selection of candidate LAGs from the HLN and WLN were similarly effective, suggesting that both LAGs and their PPIs are highly conserved. Third, we identify the inhibition of translation as a means of lifespan regulation, consistent with results from several laboratories [5], [6], [7], [39], [46]. Finally, our results demonstrate that as expected, previous screens for LAGs have not saturated the search for gene inactivations that influence longevity. This is the first study to experimentally test a large set of novel LAG predictions generated based on the concept of a longevity network. The future application of increasingly detailed biological networks to the study of aging/longevity and the potential to synergize those networks with experimental studies in *C. elegans* and other cardinal organisms are promising.

In summary, we reasoned that the first-order interacting partners of known LAGs would be more likely modulate the longevity than a random set of genes. We used previously constructed worm and human longevity networks to identify candidate lifespan regulatory genes [28]. We then narrowed this candidate gene set in a manner consistent with the principles of antagonistic pleiotropy by focusing on genes essential to development. By combining a network-based approach with the selection of genes required for development, we identified new lifespan regulatory genes at a frequency far exceeding that achieved in genome-wide screens. Though the effect of the new LAGs on lifespan is relatively modest, one can speculate that they might function in pathways or complexes that modulate core longevity functions. The interaction of genes identified in this screen with key nodes of the WLN is strongly suggestive in this regard (Figure 3). This work establishes both specifically and in proof of concept that biological networks enriched with experimental data are empowered to generate valuable candidate gene sets. Such an approach may prove broadly applicable as a tool to improve the efficiency of screening efforts. Moreover, the applicability of our method across diverse organisms or phenotypes is limited only by the availability of sufficient data to construct relevant networks.

## Supporting Information

Table S1 **First-order interactors of LAGs in the WLN (without shared genes with HLN) assayed in** ***C. elegans*** **.**
(DOCX)

Table S2 **First-order interactors of LAGs in the HLN (without shared genes with WLN) assayed in** ***C. elegans*** **.**
(DOCX)

Table S3 **Shared first-order interactors of LAGs found in WLN and HLN assayed in** ***C. elegans*** **.**
(DOCX)
