# Supplementary material for: Prediction of C. elegans Longevity Genes by Human and Worm Longevity Networks
Source: PLoS One. 2012 Oct 29;7(10):e48282. doi: 10.1371/journal.pone.0048282 (PMC3483217; doi:10.1371/journal.pone.0048282)
Supplement: Table S1 — First-order interactors of LAGs in the WLN (without shared genes with HLN) assayed in C. elegans . (DOCX) [file pone.0048282.s001.docx]

**Table S1:** First-order interactors of LAGs in the WLN (without shared genes with HLN) assayed in *C. elegans.* ^a^ Non significant vs. control.

| **Gene name** | **WormBase ID** | **Common name** | **Preliminary survival** |
| --- | --- | --- | --- |
| T20B12.7 | WBGENE00020604 | T20B12.7 | Increased |
| ZK1067.1 | WBGENE00002299 | *let-23* | Increased |
| F35G12.10 | WBGENE00000206 | *asb-1* | Increased |
| B0393.6 | WBGENE00007171 | B0393.6 | Increased |
| C27A2.6 | WBGENE00001102 | *dsh-2* | Increased |
| F09F7.4a | WBGENE00017301 | F09F7.4 | Increased |
| F47A4.2 | WBGENE00001081 | *dpy-22* | Increased |
| C45B2.7 | WBGENE00004219 | *ptr-4* | Increased |
| ZC434.6 | WBGENE00000148 | *aph-2* | Increased |
| F54D5.5 | WBGENE00010051 | F54D5.5 | Increased |
| T04A8.11 | WBGENE00011412 | T04A8.11 | Increased |
| F13G3.4 | WBGENE00008764 | *dylt-1* | Increased |
| T20B12.1 | WBGENE00020600 | T20B12.1 | Increased |
| Y75B12B.2 | WBGENE00000883 | *cyn-7* | Increased |
| C08B11.1 | WBGENE00006996 | *zyg-11* | Increased |
| C34E10.1 | WBGENE00001662 | *gop-3* | Increased |
| F43E2.7a | WBGENE00018395 | F43E2.7 | Increased |
| C47D12.2 | WBGENE00008136 | C47D12.2 | Increased |
| K10B3.7 | WBGENE00001685 | *gpd-3* | Increased |
| F09F7.3 | WBGENE00017300 | F09F7.3 | Increased |
| F37C12.9 | WBGENE00004483 | *rps-14* | Increased |
| F54E7.2 | WBGENE00004481 | *rps-12* | Increased |
| T23D8.3 | WBGENE00011944 | T23D8.3 | Increased |
| T09A5.10 | WBGENE00002994 | *lin-5* | Increased |
| C38D4.3 | WBGENE00003210 | *mel-28* | Increased |
| T20B12.8 | WBGENE00001974 | *hmg-4* | Increased |
| B0280.9 | WBGENE00015104 | B0280.9 | Increased |
| B0361.10 | WBGENE00015164 | B0361.10 | Increased |
| B0393.1 | WBGENE00004469 | *rps-0* | Increased |
| C09H10.8 | WBGENE00007502 | *glb-4* | Increased |
| C16C10.2 | WBGENE00007623 | C16C10.2 | Increased |
| C44H4.7a | WBGENE00001325 | *eor-2* | Increased |
| C45G9.5 | WBGENE00016676 | C45G9.5 | Increased |
| C54D1.5 | WBGENE00016913 | *lam-2* | Increased |
| F02D10.6 | WBGENE00008526 | F02D10.6 | Increased |
| F37C12.11 | WBGENE00004490 | *rps-21* | Increased |
| F40F8.10 | WBGENE00004478 | *rps-9* | Increased |
| F43G9.5 | WBGENE00009668 | F43G9.5 | Increased |
| F46G10.5 | WBGENE00004238 | *ptr-24* | Increased |
| K11E4.5a | WBGENE00003661 | *nhr-71* | Increased |
| M01F1.3 | WBGENE00010809 | M01F1.3 | Increased |
| R07E5.10 | WBGENE00011116 | *pdcd-2* | Increased |
| R151.3 | WBGENE00004417 | *rpl-6* | Increased |
| T01D3.1 | WBGENE00011326 | T01D3.1 | Increased |
| T02E1.3a | WBGENE00011376 | *gla-3* | Increased |
| T05A6.1 | WBGENE00000516 | *cki-1* | Increased |
| Y17G7B.15a | WBGENE00000565 | *cnt-1* | Increased |
| Y39E4B.1 | WBGENE00012714 | *abce-1* | Increased |
| ZC404.8 | WBGENE00004984 | *spn-4* | Increased |
| K07D4.3 | WBGENE00004467 | *rpn-11* | Decreased |
| R144.2a | WBGENE00020092 | R144.2 | Decreased |
| W07B3.2a | WBGENE00001561 | *gei-4* | Decreased |
| R13G10.1 | WBGENE00001086 | *dpy-27* | Decreased |
| B0285.1 | WBGENE00007135 | B0285.1 | Decreased |
| C02E7.3 | WBGENE00005245 | *srh-20* | Decreased |
| C16C10.6 | WBGENE00007627 | *ccdc-55* | Decreased |
| C16D9.2a | WBGENE00004395 | *rol-3* | Decreased |
| E03A3.3 | WBGENE00001943 | *his-69* | Decreased |
| F35G12.4a | WBGENE00009441 | F35G12.4 | Decreased |
| F57H12.1 | WBGENE00000183 | *arf-3* | Decreased |
| H20J18.1a | WBGENE00004739 | *scd-1* | Decreased |
| H38K22.2a | WBGENE00010428 | *dcn-1* | Decreased |
| K09A9.1 | WBGENE00010700 | *nipi-3* | Decreased |
| T10F2.1a | WBGENE00001744 | *grs-1* | Decreased |
| T26E3.3 | WBGENE00003921 | *par-6* | Decreased |
| W02A2.7 | WBGENE00003230 | *mex-5* | Decreased |
| Y22F5A.4 | WBGENE00003090 | *lys-1* | Decreased |
| Y49E10.14 | WBGENE00004027 | *pie-1* | Decreased |
| ZK1010.1 | WBGENE00006728 | *ubq-2* | Decreased |
| ZK1067.7 | WBGENE00004174 | *pqn-95* | Decreased |
| AH6.5 | WBGENE00003231 | *mex-6* | NS^a^ |
| B0240.3 | WBGENE00000907 | *daf-11* | NS |
| B0336.2 | WBGENE00000182 | *arf-1.2* | NS |
| B0361.6 | WBGENE00015160 | B0361.6 | NS |
| B0414.5 | WBGENE00000772 | *cpb-3* | NS |
| C01B7.4 | WBGENE00006467 | *tag-117* | NS |
| C03C10.1 | WBGENE00002202 | *kin-19* | NS |
| C04C3.3 | WBGENE00015413 | C04C3.3 | NS |
| C04H5.6 | WBGENE00003392 | *mog-4* | NS |
| C07G2.3a | WBGENE00000380 | *cct-5* | NS |
| C07H6.5 | WBGENE00000479 | *cgh-1* | NS |
| C13F10.2 | WBGENE00015742 | C13F10.2 | NS |
| C24H12.5a | WBGENE00016074 | C24H12.5 | NS |
| C26E6.3 | WBGENE00016139 | C26E6.3 | NS |
| C27F2.8 | WBGENE00016170 | C27F2.8 | NS |
| C32F10.6 | WBGENE00003601 | *nhr-2* | NS |
| C35D10.13 | WBGENE00016449 | C35D10.13 | NS |
| C35D10.5 | WBGENE00016442 | C35D10.5 | NS |
| C36A4.4 | WBGENE00007965 | C36A4.4 | NS |
| C36B1.10 | WBGENE00007977 | *gska-3* | NS |
| C38D4.6a | WBGENE00003912 | *pal-1* | NS |
| C42D4.2 | WBGENE00016595 | C42D4.2 | NS |
| C44B12.5 | WBGENE00016638 | C44B12.5 | NS |
| C55A6.9 | WBGENE00008338 | C55A6.9 | NS |
| C56C10.8 | WBGENE00002045 | *icd-1* | NS |
| EEED8.5 | WBGENE00003393 | *mog-5* | NS |
| F08F8.2 | WBGENE00017268 | F08F8.2 | NS |
| F09F7.7a | WBGENE00017304 | F09F7.7 | NS |
| F13B10.2a | WBGENE00004414 | *rpl-3* | NS |
| F22B5.7 | WBGENE00006994 | *zyg-9* | NS |
| F22G12.4 | WBGENE00009064 | F22G12.4 | NS |
| F23C8.6 | WBGENE00017735 | F23C8.6 | NS |
| F26A3.3 | WBGENE00001214 | *ego-1* | NS |
| F26E4.1 | WBGENE00006352 | *sur-6* | NS |
| F26G5.9 | WBGENE00006523 | *tam-1* | NS |
| F28D1.2 | WBGENE00009212 | F28D1.2 | NS |
| F32A11.2 | WBGENE00001998 | *hpr-17* | NS |
| F32H2.3 | WBGENE00004953 | *spd-2* | NS |
| F33E11.2 | WBGENE00018010 | F33E11.2 | NS |
| F35G12.8 | WBGENE00004874 | *smc-4* | NS |
| F37C12.13a | WBGENE00018154 | *exos-9* | NS |
| F38A6.3a | WBGENE00001851 | *hif-1* | NS |
| F38E1.7 | WBGENE00003395 | *mom-2* | NS |
| F38H4.8 | WBGENE00001151 | *ech-2* | NS |
| F40F11.2 | WBGENE00009587 | F40F11.2 | NS |
| F45F2.12 | WBGENE00001882 | *his-8* | NS |
| F54C8.3 | WBGENE00001284 | *emb-30* | NS |
| F54D5.9 | WBGENE00010053 | F54D5.9 | NS |
| F55A8.1 | WBGENE00001186 | *egl-18* | NS |
| F56A11.3 | WBGENE00001165 | *efn-4* | NS |
| F56D12.5a | WBGENE00006924 | *vig-1* | NS |
| F56F3.2a | WBGENE00003577 | *ndg-4* | NS |
| F57B10.12 | WBGENE00003184 | *mei-2* | NS |
| F58F6.4 | WBGENE00004338 | *rfc-2* | NS |
| F58G11.1a | WBGENE00010279 | *letm-1* | NS |
| F59A2.4 | WBGENE00010304 | F59A2.4 | NS |
| H02I12.5 | WBGENE00010353 | H02I12.5 | NS |
| H04J21.3a | WBGENE00001589 | *gip-1* | NS |
| K04C1.5 | WBGENE00010555 | K04C1.5 | NS |
| K04G7.1 | WBGENE00019400 | K04G7.1 | NS |
| K10D2.6 | WBGENE00001262 | *emb-8* | NS |
| M176.6a | WBGENE00002199 | *kin-15* | NS |
| M88.2 | WBGENE00010905 | *m88.2* | NS |
| M88.6a | WBGENE00003915 | *pan-1* | NS |
| R07E5.14 | WBGENE00004387 | *rnp-4* | NS |
| R10E4.4 | WBGENE00003157 | *mcm-5* | NS |
| T01G9.5a | WBGENE00003183 | *mei-1* | NS |
| T04A8.7a | WBGENE00011409 | T04A8.7 | NS |
| T05C12.6a | WBGENE00003241 | *mig-5* | NS |
| T10F2.4 | WBGENE00020423 | T10F2.4 | NS |
| T11F8.3 | WBGENE00004374 | *rme-2* | NS |
| T22A3.3 | WBGENE00003083 | *lst-1* | NS |
| T24D1.3 | WBGENE00011986 | T24D1.3 | NS |
| VF36H2L.1 | WBGENE00000147 | *aph-1* | NS |
| Y48B6A.2 | WBGENE00004456 | *rpl-43* | NS |
| Y54E2A.3 | WBGENE00006381 | *tac-1* | NS |
| Y56A3A.21 | WBGENE00013238 | *trap-4* | NS |
| ZC155.3 | WBGENE00022531 | *morc-1* | NS |
| ZK1037.5 | WBGENE00014193 | *nhr-247* | NS |
