# Supplementary material for: Prediction of C. elegans Longevity Genes by Human and Worm Longevity Networks
Source: PLoS One. 2012 Oct 29;7(10):e48282. doi: 10.1371/journal.pone.0048282 (PMC3483217; doi:10.1371/journal.pone.0048282)
Supplement: Table S2 — First-order interactors of LAGs in the HLN (without shared genes with WLN) assayed in C. elegans . (DOCX) [file pone.0048282.s002.docx]

**Table S2:** First-order interactors of LAGs in the HLN (without shared genes with WLN) assayed in *C. elegans.* ^a^ Non significant vs. control.

| **Gene name** | **WormBase ID** | **Common name** | **Preliminary survival** |
| --- | --- | --- | --- |
| C09G12.9 | WBGENE00015658 | tsg-101 | Increased |
| R01H10.8 | WBGENE00000564 | cnk-1 | Increased |
| R08D7.3 | WBGENE00001227 | eif-3.d | Increased |
| T05G5.3 | WBGENE00000405 | cdk-1 | Increased |
| H39E23.1a | WBGENE00003916 | par-1 | Increased |
| F22B5.1 | WBGENE00001358 | evl-20 | Increased |
| R11D1.8 | WBGENE00004442 | rpl-28 | Increased |
| T10G3.6 | WBGENE00001808 | gut-2 | Increased |
| Y39G8C.1 | WBGENE00012730 | xrn-1 | Increased |
| F59E10.1 | WBGENE00003882 | orc-2 | Increased |
| T09B4.10 | WBGENE00000500 | chn-1 | Increased |
| C01H6.5a | WBGENE00003622 | nhr-23 | Increased |
| F31D4.3 | WBGENE00001431 | fkb-6 | Increased |
| F29G9.3 | WBGENE00000159 | aps-1 | Increased |
| R02D3.3 | WBGENE00019821 | R02D3.3 | Increased |
| F10B5.6 | WBGENE00001281 | emb-27 | Increased |
| F18A1.5 | WBGENE00017546 | rpa-1 | Increased |
| F28D9.1 | WBGENE00004706 | rsr-1 | Increased |
| B0412.4 | WBGENE00004498 | rps-29 | Increased |
| B0464.1 | WBGENE00001094 | drs-1 | Increased |
| B0511.10 | WBGENE00001228 | eif-3.e | Increased |
| B0547.1 | WBGENE00000817 | csn-5 | Increased |
| C18D11.4 | WBGENE00004705 | rsp-8 | Increased |
| C30C11.4 | WBGENE00016250 | C30C11.4 | Increased |
| C34E10.2 | WBGENE00001661 | gop-2 | Increased |
| C47D12.8 | WBGENE00008140 | C47D12.8 | Increased |
| C54G10.2 | WBGENE00004337 | rfc-1 | Increased |
| C56C10.10 | WBGENE00016966 | C56C10.10 | Increased |
| F10C2.4 | WBGENE00008645 | F10C2.4 | Increased |
| F11C1.6a | WBGENE00003623 | nhr-25 | Increased |
| F16D3.1 | WBGENE00006531 | tba-5 | Increased |
| F26B1.2a | WBGENE00017816 | F26B1.2 | Increased |
| F32A5.7 | WBGENE00003078 | lsm-4 | Increased |
| F32D1.10 | WBGENE00003159 | mcm-7 | Increased |
| R01H10.1 | WBGENE00001002 | div-1 | Increased |
| R11A8.6 | WBGENE00002152 | irs-1 | Increased |
| T02G5.9a | WBGENE00002238 | krs-1 | Increased |
| T05H4.5 | WBGENE00020268 | T05H4.5 | Increased |
| T23G7.1 | WBGENE00001061 | dpl-1 | Increased |
| T28F12.3 | WBGENE00004947 | sos-1 | Increased |
| Y38F1A.5 | WBGENE00000870 | cyd-1 | Increased |
| ZK1098.1 | WBGENE00014218 | ZK1098.1 | Increased |
| ZK131.3 | WBGENE00001883 | his-9 | Increased |
| ZK328.5b | WBGENE00003796 | npp-10 | Increased |
| ZK652.1 | WBGENE00004918 | snr-5 | Increased |
| ZK669.1a | WBGENE00014051 | tag-341 | Increased |
| K02D10.5 | WBGENE00019305 | K02D10.5 | Decreased |
| D1014.3 | WBGENE00017016 | snap-1 | Decreased |
| F26H9.6 | WBGENE00004268 | rab-5 | Decreased |
| ZK1058.2 | WBGENE00003930 | pat-3 | Decreased |
| C52E4.4 | WBGENE00004501 | rpt-1 | Decreased |
| Y40B1A.4 | WBGENE00012735 | sptf-3 | Decreased |
| C23G10.4a | WBGENE00004459 | rpn-2 | Decreased |
| C39F7.4 | WBGENE00004266 | rab-1 | Decreased |
| F09E5.1 | WBGENE00004034 | pkc-3 | Decreased |
| F16D3.4 | WBGENE00008887 | F16D3.4 | Decreased |
| F22B3.2 | WBGENE00001937 | his-63 | Decreased |
| F36A4.7 | WBGENE00000123 | ama-1 | Decreased |
| F38E11.5 | WBGENE00009542 | F38E11.5 | Decreased |
| F38H4.9 | WBGENE00002363 | let-92 | Decreased |
| F45F2.13 | WBGENE00001880 | his-6 | Decreased |
| F46F2.2a | WBGENE00002203 | kin-20 | Decreased |
| F48E8.5 | WBGENE00003901 | paa-1 | Decreased |
| F49C12.8 | WBGENE00004463 | rpn-7 | Decreased |
| F54D5.11 | WBGENE00010054 | F54D5.11 | Decreased |
| F54E12.1 | WBGENE00001929 | his-55 | Decreased |
| R10E11.1a | WBGENE00000366 | cbp-1 | Decreased |
| W04A8.7 | WBGENE00006382 | taf-1 | Decreased |
| W09B6.1a | WBGENE00004076 | pod-2 | Decreased |
| Y18D10A.5 | WBGENE00001746 | gsk-3 | Decreased |
| Y41E3.11 | WBGENE00012769 | Y41E3.11 | Decreased |
| Y6B3A.1a | WBGENE00012386 | agef-1 | Decreased |
| ZK742.1a | WBGENE00002078 | xpo-1 | Decreased |
| B0035.12 | WBGENE00007111 | B0035.12 | NS^a^ |
| B0304.1a | WBGENE00001948 | hlh-1 | NS |
| B0336.6 | WBGENE00015146 | abi-1 | NS |
| B0414.7a | WBGENE00003472 | mtk-1 | NS |
| C07A12.4a | WBGENE00003963 | pdi-2 | NS |
| C07E3.2 | WBGENE00007413 | pro-2 | NS |
| C08B11.5 | WBGENE00004723 | sap-49 | NS |
| C09G4.5 | WBGENE00003224 | mes-6 | NS |
| C10H11.9 | WBGENE00002694 | let-502 | NS |
| C14B1.5 | WBGENE00007576 | C14B1.5 | NS |
| C14B9.4a | WBGENE00004042 | plk-1 | NS |
| C28H8.6a | WBGENE00016197 | pxl-1 | NS |
| C31E10.7 | WBGENE00007848 | C31E10.7 | NS |
| C32E8.5 | WBGENE00016323 | C32E8.5 | NS |
| C32F10.2 | WBGENE00003020 | lin-35 | NS |
| C36B1.3 | WBGENE00007971 | rpb-3 | NS |
| C37F5.1 | WBGENE00002990 | lin-1 | NS |
| C39E9.14a | WBGENE00001007 | dli-1 | NS |
| C41D11.2 | WBGENE00001231 | EIF-3.H | NS |
| C43E11.10 | WBGENE00000382 | cdc-6 | NS |
| C47E12.5 | WBGENE00006699 | uba-1 | NS |
| C48D5.1 | WBGENE00003605 | nhr-6 | NS |
| C48E7.6 | WBGENE00016751 | C48E7.6 | NS |
| C50F2.3 | WBGENE00016837 | C50F2.3 | NS |
| C50H2.1 | WBGENE00008239 | fshr-1 | NS |
| C52E4.3 | WBGENE00004917 | snr-4 | NS |
| D1037.4 | WBGENE00004272 | rab-8 | NS |
| D2089.1a | WBGENE00004704 | rsp-7 | NS |
| F01G4.1 | WBGENE00004204 | psa-4 | NS |
| F08G2.3 | WBGENE00001916 | his-42 | NS |
| F10B5.1 | WBGENE00004421 | rpl-10 | NS |
| F10B5.5 | WBGENE00008641 | pch-2 | NS |
| F10G8.3 | WBGENE00003803 | npp-17 | NS |
| F14B4.2a | WBGENE00008780 | F14B4.2 | NS |
| F14D12.2 | WBGENE00006826 | unc-97 | NS |
| F16B4.8 | WBGENE00000387 | cdc-25.2 | NS |
| F17E9.10 | WBGENE00001906 | his-32 | NS |
| F18G5.3 | WBGENE00001674 | gpa-12 | NS |
| F23B12.7 | WBGENE00009084 | F23B12.7 | NS |
| F25H5.5 | WBGENE00009127 | F25H5.5 | NS |
| F28B3.7 | WBGENE00001860 | him-1 | NS |
| F28D1.10 | WBGENE00001580 | gex-3 | NS |
| F29B9.6 | WBGENE00006706 | ubc-9 | NS |
| F32A6.3 | WBGENE00017974 | vps-41 | NS |
| F33D4.1a | WBGENE00003607 | nhr-8 | NS |
| F37D6.1 | WBGENE00009507 | mus-101 | NS |
| F37E3.1 | WBGENE00018156 | ncbp-1 | NS |
| F42E11.4 | WBGENE00006584 | tni-1 | NS |
| F43C1.2a | WBGENE00003401 | mpk-1 | NS |
| F45H11.4 | WBGENE00003233 | mgl-2 | NS |
| F52E4.7 | WBGENE00018703 | sec-3 | NS |
| F54E12.2 | WBGENE00010061 | F54E12.2 | NS |
| F55F8.4 | WBGENE00018892 | cir-1 | NS |
| F55G1.2 | WBGENE00001933 | his-59 | NS |
| F57B10.6 | WBGENE00019004 | xpg-1 | NS |
| F57F5.1 | WBGENE00010204 | F57F5.1 | NS |
| F59G1.5 | WBGENE00004214 | ptp-2 | NS |
| H43I07.2 | WBGENE00019275 | H43I07.2 | NS |
| K01G5.4 | WBGENE00004302 | ran-1 | NS |
| K01H12.2 | WBGENE00010485 | ant-1.3 | NS |
| K02B12.1 | WBGENE00000431 | ceh-6 | NS |
| K04D7.1 | WBGENE00010556 | rack-1 | NS |
| K08F8.6 | WBGENE00002295 | let-19 | NS |
| M01D7.7a | WBGENE00001196 | egl-30 | NS |
| M03D4.1a | WBGENE00006974 | zen-4 | NS |
| M04F3.1 | WBGENE00019767 | rpa-2 | NS |
| R02D3.5 | WBGENE00019823 | R02D3.5 | NS |
| R06F6.2 | WBGENE00011067 | vps-11 | NS |
| R09B3.1 | WBGENE00001372 | exo-3 | NS |
| R11A5.1a | WBGENE00000163 | apb-3 | NS |
| R74.1 | WBGENE00003073 | lrs-1 | NS |
| T03E6.7 | WBGENE00000776 | cpl-1 | NS |
| T04H1.4 | WBGENE00004296 | rad-50 | NS |
| T10C6.12 | WBGENE00001877 | his-3 | NS |
| T11G6.1a | WBGENE00002001 | hrs-1 | NS |
| T12D8.1 | WBGENE00011729 | set-16 | NS |
| T13H5.4 | WBGENE00011758 | T13H5.4 | NS |
| T14F9.1 | WBGENE00020507 | vha-15 | NS |
| T14G10.5 | WBGENE00011775 | T14G10.5 | NS |
| T16H12.4 | WBGENE00011814 | T16H12.4 | NS |
| T20G5.2 | WBGENE00000833 | cts-1 | NS |
| T23D8.6 | WBGENE00001942 | his-68 | NS |
| T24H7.1 | WBGENE00004015 | phb-2 | NS |
| T27E9.3 | WBGENE00000407 | cdk-5 | NS |
| T27F2.1 | WBGENE00004806 | skp-1 | NS |
| T28D9.10 | WBGENE00004916 | snr-3 | NS |
| VC5.4 | WBGENE00007029 | mys-1 | NS |
| W02D3.9 | WBGENE00006773 | unc-37 | NS |
| W02F12.5 | WBGENE00020950 | W02F12.5 | NS |
| W03B1.4 | WBGENE00005662 | srs-1 | NS |
| W07E6.4 | WBGENE00004188 | prp-21 | NS |
| W08E3.1 | WBGENE00004915 | snr-2 | NS |
| W09C5.2 | WBGENE00006793 | unc-59 | NS |
| W10D5.3a | WBGENE00001574 | gei-17 | NS |
| Y116A8C.32 | WBGENE00013808 | sfa-1 | NS |
| Y116A8C.42 | WBGENE00004914 | snr-1 | NS |
| Y47D3B.7 | WBGENE00004735 | sbp-1 | NS |
| Y48B6A.3 | WBGENE00006964 | xrn-2 | NS |
| Y49E10.15 | WBGENE00004919 | snr-6 | NS |
| Y56A3A.20 | WBGENE00000369 | ccf-1 | NS |
| Y56A3A.32 | WBGENE00006937 | wah-1 | NS |
| Y66H1B.3 | WBGENE00022049 | Y66H1B.3 | NS |
| ZC395.10 | WBGENE00022599 | ZC395.10 | NS |
| ZC410.2 | WBGENE00013880 | mppb-1 | NS |
| ZK1127.4 | WBGENE00022851 | ZK1127.4 | NS |
| ZK1128.5 | WBGENE00044072 | TAG-246 | NS |
| ZK131.2 | WBGENE00001899 | his-25 | NS |
| ZK131.7 | WBGENE00001887 | his-13 | NS |
| ZK20.5 | WBGENE00004468 | rpn-12 | NS |
| ZK507.6 | WBGENE00013981 | ZK507.6 | NS |
| ZK546.15 | WBGENE00006619 | try-1 | NS |
| ZK550.4 | WBGENE00013998 | ZK550.4 | NS |
