# Supplementary material for: Prediction of C. elegans Longevity Genes by Human and Worm Longevity Networks
Source: PLoS One. 2012 Oct 29;7(10):e48282. doi: 10.1371/journal.pone.0048282 (PMC3483217; doi:10.1371/journal.pone.0048282)
Supplement: Table S3 — Shared first-order interactors of LAGs found in WLN and HLN assayed in C. elegans . (DOCX) [file pone.0048282.s003.docx]

**Table S3:** Shared first-order interactors of LAGs found in WLN and HLN assayed in *C. elegans.* ^a^ Non significant vs. control.

| **Gene name** | **WormBase ID** | **Common name** | **Preliminary survival** |
| --- | --- | --- | --- |
| F58A3.2a | WBGENE00001184 | egl-15 | Increased |
| C14A4.11 | WBGENE00007561 | C14A4.11 | Increased |
| C03C10.3 | WBGENE00004392 | rnr-2 | Increased |
| F26F4.11 | WBGENE00017830 | rpb-8 | Increased |
| C09D8.1a | WBGENE00004215 | ptp-3 | Increased |
| F26F4.10a | WBGENE00004679 | rrt-1 | Increased |
| K04G2.8a | WBGENE00000156 | apr-1 | Increased |
| T20B12.2 | WBGENE00006542 | tbp-1 | Increased |
| T20B12.3 | WBGENE00020601 | T20B12.3 | Increased |
| F32E10.4 | WBGENE00002074 | ima-3 | Decreased |
| F47D12.4a | WBGENE00001972 | hmg-1.2 | Decreased |
| T01G9.6a | WBGENE00002196 | kin-10 | Decreased |
| T08A11.2 | WBGENE00011605 | T08A11.2 | Decreased |
| T10C6.13 | WBGENE00001876 | his-2 | Decreased |
| W01B6.9 | WBGENE00003576 | ndc-80 | Decreased |
| ZC302.1 | WBGENE00003405 | mre-11 | Decreased |
| AC7.2a | WBGENE00004929 | soc-2 | NS^a^ |
| B0414.2 | WBGENE00004393 | rnt-1 | NS |
| C06C3.1a | WBGENE00003196 | mel-11 | NS |
| C10F3.5 | WBGENE00003954 | pcm-1 | NS |
| C26E6.4 | WBGENE00016140 | rpb-2 | NS |
| C27H6.2 | WBGENE00007784 | ruvb-1 | NS |
| C36E8.5 | WBGENE00006537 | tbb-2 | NS |
| C37A2.4a | WBGENE00000871 | cye-1 | NS |
| C53A5.3 | WBGENE00001834 | hda-1 | NS |
| F10C5.1 | WBGENE00003134 | mat-3 | NS |
| F25H2.5 | WBGENE00009119 | F25H2.5 | NS |
| F39B2.4a | WBGENE00006349 | sur-2 | NS |
| F54E7.3a | WBGENE00003918 | par-3 | NS |
| F58A4.8 | WBGENE00006540 | tbg-1 | NS |
| F59A2.1a | WBGENE00003795 | npp-9 | NS |
| H14A12.2a | WBGENE00001503 | fum-1 | NS |
| K05C4.6 | WBGENE00001979 | hmp-2 | NS |
| K07A1.12 | WBGENE00003036 | lin-53 | NS |
| K08B4.1a | WBGENE00002245 | lag-1 | NS |
| K12C11.2 | WBGENE00004888 | smo-1 | NS |
| M03A1.1a | WBGENE00006868 | vab-1 | NS |
| R07E5.3 | WBGENE00011111 | R07E5.3 | NS |
| W04D2.1a | WBGENE00000228 | atn-1 | NS |
| W08F4.8 | WBGENE00021097 | cdc-37 | NS |
| Y43C5A.6a | WBGENE00004297 | rad-51 | NS |
| Y57G11C.24a | WBGENE00001330 | eps-8 | NS |
| Y66H1B.2a | WBGENE00022048 | Y66H1B.2 | NS |
